# Reducing Racial Inequities in Health: Using What We Already Know to Take Action

**DOI:** 10.3390/ijerph16040606

**Published:** 2019-02-19

**Authors:** David R. Williams, Lisa A. Cooper

**Affiliations:** 1 Department of Social and Behavioral Sciences, Harvard T.H. Chan School of Public Health, Boston, MA 02115, USA; 2 Department of African and African American Studies and of Sociology, Harvard University, Cambridge, MA 02138, USA; 3 Department of Psychiatry and Mental Health, University of Cape Town, Groote Schuur Hospital Observatory, Cape Town 7925, South Africa; 4 Department of Medicine, Johns Hopkins University School of Medicine, Baltimore, MD 21287, USA; lisa.cooper@jhmi.edu; 5 Department of Health, Behavior and Society, Bloomberg School of Public Health, Johns Hopkins University, Baltimore, MD 21205, USA

**Keywords:** race, racism, ethnicity, inequities, disparities, interventions

## Abstract

This paper provides an overview of the scientific evidence pointing to critically needed steps to reduce racial inequities in health. First, it argues that communities of opportunity should be developed to minimize some of the adverse impacts of systemic racism. These are communities that provide early childhood development resources, implement policies to reduce childhood poverty, provide work and income support opportunities for adults, and ensure healthy housing and neighborhood conditions. Second, the healthcare system needs new emphases on ensuring access to high quality care for all, strengthening preventive health care approaches, addressing patients’ social needs as part of healthcare delivery, and diversifying the healthcare work force to more closely reflect the demographic composition of the patient population. Finally, new research is needed to identify the optimal strategies to build political will and support to address social inequities in health. This will include initiatives to raise awareness levels of the pervasiveness of inequities in health, build empathy and support for addressing inequities, enhance the capacity of individuals and communities to actively participate in intervention efforts and implement large scale efforts to reduce racial prejudice, ideologies, and stereotypes in the larger culture that undergird policy preferences that initiate and sustain inequities.

## 1. Introduction

Large racial and ethnic (in the interest of parsimony, we use the term ‘racial’ to describe both) inequities in health in the U.S. highlight the need for renewed efforts to effectively reduce and eliminate them. The historically stigmatized racial groups, blacks (or African Americans), Native Americans (or American Indians and Alaska Natives) and Native Hawaiians and Other Pacific Islanders, have worse health than that of whites [1], and despite progress in reducing inequities over time, racial gaps in health persist. For example, the white advantage over blacks in life expectancy at birth declined from 8.3 years in 1950 to 3.7 years in 2016 [2], and although we lack life expectancy data on Native Americans for 1950, American Indians currently have lower life expectancy than African Americans [3]. The health profiles for Asians and Hispanics (or Latinos) are influenced by the high proportion of immigrants within these populations. Immigrants of all racial groups tend to have lower mortality rates than their native-born peers but their health advantage declines with increasing length of stay in the U.S. For example, in one national study, middle-aged U.S.-born Mexican Americans and Mexican immigrants resident 20 or more years in the U.S. had a health profile that did not differ from that of African Americans, while recent immigrants had a health profile similar to whites [4]. Importantly, part of the narrowing of the black-white gap in health over time may be an artifact of changes in the composition of the black population. Black immigrants are an increasing share of the black population and black migration from Africa has outpaced migration from the Caribbean since 2000, with African immigrants experiencing smaller declines in health than their Caribbean counterparts with increasing length of stay in the U.S. [5].

Current child health data provide reason for concern about potential increases in racial inequities for both physical and mental health outcomes in the future. For example, a recent study indicates that black and Hispanic children are more likely to be obese than their white peers at age 2 and these disparities persist into adulthood [6]. That is, two-thirds of blacks and Hispanics currently aged 2 to 19, are projected to be obese by the age 35, compared to the national average of 57% [6]. Obesity is a risk factor for several chronic conditions and these data suggest that there are likely to be disparities for multiple major causes of death in the future. National data on suicide trends among elementary school children also provide cause for concern. A recent study found that between 1993 and 2012, among children aged 5 to 11 years, the overall suicide rate for this age group was stable, obscuring that while the rate had declined for whites and was stable for Hispanics and other racial groups, it had almost doubled for blacks [7].

This article provides an overview of the available scientific evidence that points to three areas of needed intervention to reduce and ultimately eliminate racial inequities in health. First, comprehensive efforts are needed to create and maintain opportunities that facilitate health and its determinants at the level of the local community. Second, health care providers and institutions should give greater emphasis to prevention, address patients’ social risk factors and needs and ensure that every client receives appropriate, high quality care. Third, major new investments are needed to inform the public and policymakers about the nature and extent of racial inequities in health and to enhance individual and community capacity and build public empathy and political will to effectively address them.

## 2. Strategy Number One: Creating Communities of Opportunity

Reducing inequities in health requires dismantling the systems that initiate and sustain inequities in a broad range of societal institutions that are the drivers of inequities in health. All of these societal inequities are driven by racism. Racism is an organized societal system, in which the dominant racial group, based on a hierarchy of human value, categorizes and ranks people into social groups called “races”, and uses its power to devalue, disempower, and differentially allocate societal resources and opportunities to groups defined as inferior [8,9]. As a structured system, racism interacts with other social institutions, such as the political, legal, and economic institutions, shaping the values, policies and practices within these institutions and being re-shaped by them. By creating unequal access to resources and opportunity, racism is a fundamental cause of racial inequities in health [10,11].

Reskin has emphasized that racism has created a set of dynamic, interdependent, components or subsystems that reinforce each other and create and sustain reciprocal causality of racial inequities across various sectors of society [12]. Moreover, the processes creating these inequities are dynamic and interrelated such that racial inequities in any given societal domain are a product of racist policies and processes across multiple domains and subsystems [12]. Thus, structural or institutional racism (we use these terms interchangeably) exists within, and is reinforced and supported by multiple societal systems, including the housing market, the education system, the labor market, the criminal justice system, credit markets, the economy and the health care system. The bottom-line is that that the system of racism has created reduced access for stigmatized groups to the many opportunities that facilitate socioeconomic attainment, quality of life and health. To neutralize these negative effects and build healthier and more equitable communities, we call for investments to create “communities of opportunity”. We use this term to describe the transformation of local communities (that had been historically disadvantaged because of racism and its related systematic under-investments), into places that provide opportunities in education, labor markets, housing markets, credit markets, health care and all other domains that drive well-being. We view the underlying existing inequities as products of racism and the creation of communities of opportunity as a systematic, comprehensive and coordinated national initiative to eliminate the racism that is embedded in policies, procedures and the routine operation of many societal institutions. Improving health of disadvantaged groups and reducing gaps in health requires changing systems to improve conditions that determine health in homes, schools, neighborhoods, workplaces, houses of worship and other social contexts. Given the multi-faceted and systemic nature of racism, the specific initiatives described below are inter-related and need to build on each other to neutralize the adverse effects of racism.

### 2.1. Early Childhood Development Initiatives

Inequities in health begin early in life and effectively reducing them calls for investments in early childhood interventions. Research reveals that starting early can have dramatic life-long positive impacts on health and the social determinants of health. The Perry Preschool Program was a two-year school-based early childhood intervention program in which African American 3 to 4 year olds from a public housing project in Ypsilanti, Michigan were randomized to receive the intervention or to be in a control group [13]. This intervention consisted of morning sessions at school and afternoon home visits by the teacher. At age 10, children who received the intervention did not have higher IQ scores than the children in the control condition, but they had higher achievement test scores suggesting that they had greater motivation for learning [14]. At age 40, the intervention group had higher income, high school completion, college graduation, health insurance coverage and home ownership and lower rates of crime, out-of-wedlock births, and welfare assistance compared to the controls [13]. At age 40, the intervention group also had better overall health and engaged in fewer risky behaviors (driving without seat belt, smoking, illicit use of sedatives, marijuana, LSD, cocaine, heroin) although there were no differences in medical conditions [13].

The Abecedarian project based in Chapel Hill, North Carolina is another early childhood intervention program. It began in 1972 and randomized infants from poor households (mainly African American) to an early childhood intervention that provided services from birth to age 5. The program provided a safe and nurturing environment with cognitive and social stimulation (language development, emotional regulation and cognitive skills), access to pediatric care, good nutrition, and caregiving and supervised play for 8 hours per day. By age 21, individuals in the intervention group had fewer symptoms of depression, lower marijuana use, a more active lifestyle, and significant educational and vocational advantages compared to the controls [15,16]. By their mid-30s, the children who received the intervention had lower levels of multiple risk factors of cardiovascular and metabolic disease with the effects being stronger for males than for females [17]. For example, males in the treatment group had a systolic blood pressure of 126 millimeters of mercury (mm, Hg) compared to 143 mm Hg in the control group. Similarly, none of the males in the treatment group, compared to one in four in the control group, met criteria for metabolic syndrome. The study also found that both males and females in the treatment group had significantly lower scores on the Framingham Risk score for coronary heart disease. Economic analyses reveal that early childhood programs have a net return to society of $3 to $17 for each dollar invested [18].

There is considerable variation in the quality of early childhood development programs, but the available scientific evidence indicates that large positive impacts of these programs are only evident for those that are high in quality [19]. Moreover, to achieve the largest societal impact, effective interventions should be provided during the prenatal period and in the first three years of life, maximizing access to the children and families who are the most disadvantaged [19].

### 2.2. Reducing Childhood Poverty

Communities of opportunity should also use policy levers to build resilience for children through policies that reduce childhood poverty. Poverty is not distributed equally across racial groups in the United States. In 2017, 33% of American Indian children, 33% of African American children, 26% of Hispanic children, 11% of Asian and Pacific Islander children, and 11% of white children lived in households below the poverty line [20]. Research reveals that children who grow up poor are at substantially elevated risk of reduced socioeconomic status (SES) long-term. On average, adults who were raised in poor families complete fewer years of school, earn lower incomes, and are more likely to be poor in adulthood relative to adults who do not come from a poor family [21]. In international comparisons, the U.S. has high rates of childhood poverty compared to other wealthy countries. Importantly, other countries do a much better job at reducing childhood poverty than the U.S. Data from the United Nations International Children’s Emergency Fund (UNICEF) illustrate this [22]. It shows, for example that the child poverty rate in Australia, before taxes and transfers, was 28% but was reduced to 12% after taxes and transfers. Similarly, child poverty in Canada was reduced from 25% before taxes and transfers to 13% after taxes and transfers. In striking contrast, in the U.S., the child poverty rate was reduced from 24% before taxes and transfers to 23% after them. This illustrates that the economies of some of our peer nations produce child poverty rates similar to the U.S., but those societies have developed policies to markedly reduce child poverty. Taxes and transfers are policy preferences that reflect the values of a society (such as enhancing family income or providing supplementary income, nutrition or housing) and these striking international comparisons illustrate the opportunities that exist in the U.S. to implement polices to improve the economic well-being of America’s children.

More generally, other research indicates that states that had more generous policies supporting the well-being of vulnerable populations had better health. For example, a study that analyzed cross-sectional data for all 50 states for a 10-year period found that U.S. states with higher spending on education, greater public expenditures, less regressive taxes, and more generous welfare policies (as reflected in Temporary Assistance to Needy Families (TANF) and Medicaid program rules) had better health as measured by lower death rates, with the effects being stronger for overall mortality than for infant mortality [23]. The study found that every $100 increase in spending on public education led to two fewer deaths per 100,000 population and a one standard deviation change in tax progressivity resulted in 6 fewer deaths per 100,000 population. In comparison, a one percent increase in smoking and obesity rates was associated with an increase of one and two deaths per 100,000 population, respectively. Future research needs to better understand the specific aspects of social welfare systems that matter for health.

### 2.3. Enhancing Income and Employment Opportunities among Youth and Adults

Communities of opportunity also need to ensure that everyone has access to employment opportunities that ensure an adequate income to support health. Research reveals that social policies that provide families with additional income can lead to improved health. For example, the Earned Income Tax Credit (EITC) is a government cash transfer program that provides a cash award via the tax system to low income working families in the U.S. A study using variation in the federal EITC over time and the presence of state EITC’s found that income from EITC reduced the rate of low birth weight and increased mean birth weight, with the associations being larger for blacks than for whites [24]. Low birth weight is a leading cause of mortality among newborns and is associated with an increased risk of health problems in infancy, childhood and adulthood. Another study analyzed changes in state EITC as a natural experiment and found that state EITCs increased birth weights and reduced maternal smoking [25].

Similarly, a recent study found that increases in the minimum wage were associated with improved birth outcomes [26]. This study used a quasi-experimental difference-in-difference research design to examine the effects of state-level minimum wage for each of the 50 states, by month, from 1980-2011. It found that a dollar increase in the minimum wage above the federal minimum was associated with a 1% to 2% decrease in low birth weight and a 4% decrease in post-neonatal mortality. The researchers estimated that if all states in 2014 had increased their minimum wage by one dollar, there would have been 2790 fewer low birth weight births and 518 fewer post-neonatal deaths for the year. Income supplementation is also associated with improved health of the elderly. The Social Security program is an old-age pension program that provides additional income to the elderly. Research reveals that both the initial implementation of the program and later increases in the level of benefits were associated with mortality declines for the elderly [27].

Some limited research also indicates that income supplementation to racial minorities can lead to improved health and the reduction of at least some racial differences in health. The Great Smoky Mountain Study in North Carolina was a natural experiment that assessed the impact of additional income on the health of American Indian youth who were to 9 to 13 years old at baseline [28]. During the course of this longitudinal study, Native American households received extra income due to the opening of a Casino. The study found declining rates of deviant and aggressive behavior among adolescents whose families received additional income, with the level of psychiatric symptoms for those who had received the cash supplements for four years being similar to those of adolescents who had never been poor [28]. Moreover, this lower risk of psychiatric disorders in adolescence when the youth lived at home persisted into young adulthood when most had established their own residence [29]. Importantly, subgroup analyses revealed that this effect was present only for those in the youngest cohort (age 12 when the supplements began) who had had the longest exposure to the additional income with no effect of income evident in the two older cohorts who were age 14 and age 16 at the time of the initial supplement. Other analyses revealed that the additional income received by adolescents was associated with increases in education and reductions in minor criminal offenses for Native American youth and the elimination of the American Indian- white disparities for both of these outcomes [30]. These effects were driven by improvements for those adolescents who were poor at the time of the inception of income supplements.

Civil rights policies are an example of a large-scale economic initiative that led to improved health for blacks and reduced black-white disparities in health. Research reveals that civil rights policies and their enforcement in education and employment reduced racial inequities [31]. For example, civil rights policies led to increases in employment and wages that narrowed the black-white economic gap with the gains being larger for black women than for black men [32]. One study documented that during 1965–1974 black women had larger gains in life expectancy than black men and white men and women, with the life expectancy increase for black women during this period being three times as large as those in the decade before [32]. Another study found that between 1968 and 1978, both black males and females, aged 35-74, had larger absolute and relative declines in mortality than their white counterparts, with the mortality declines being larger for black women than black men [33]. Other data reveal that black women born between 1967 and 1969 had better health status as adults and were less likely to have low-birth weight infants and infants with low APGAR scores than those born between 1961 and 1963 [34]. Civil Rights policies and their enforcement in health care also led to improvements in health for blacks [31]. For example, the desegregation of southern hospitals enabled 5,000 to 7,000 additional black babies to survive infancy between 1965–1975 [35]. Thus, the mid 1960s to the mid 1970s was a period in which the health of African Americans improved more rapidly than the health of whites so that there was a narrowing of the black-white gap in health. At the same time, the failure to enforce civil rights policies in housing has significantly contributed to the persistence of racial gaps in economic status [31]. Rigorous empirical analyses reveal that ending racial residential segregation would completely eliminate racial differences, among young adults, in income, education, and unemployment and reduce gaps in single motherhood by two-thirds [36].

Research also reveals that improved economic well-being of adults appears to trigger ripple effects that include improved family relationships. In the Great Smoky Mountain Study researchers concluded that the underlying driver of the observed heath impacts of additional income was improved parenting [30]. Improved economic circumstances can also reduce the prevalence of single parent households. Research on military enlistment indicates that it has positive effects on family formation and stability. Military enlistment is an example of a policy that leads to socioeconomic status (SES) advancement. The military is a more race-blind environment than the larger society and military service has provided disadvantaged black men increased education, higher earnings and greater occupational mobility than their civilian peers [37]. Military benefits include resources that support maintaining a family: family housing, day care centers, and school-age activity centers. This economic mobility is likely to have positive effects on the health of the adults in military households and lead to higher academic achievement and better health for their children. Research reveals that active duty military service promotes marriage over cohabitation, increases the likelihood of first marriage, and leads to greater stability of marriage and all of these effects were greater for blacks than for whites [37,38,39]. These data suggest that stable economic resources and trajectories of opportunity and hope for low-income black males could reduce and potentially even eliminate racial disparities in marriage.

Such initiatives could markedly reduce the negative effects of household structure on child development and well-being. Research finds that being raised in a single-parent household has adverse impacts on multiple outcomes. For example, children raised in single-parent homes perform more poorly in school, and are less likely to graduate from high school [40]. They are also more likely to become sexually active at a younger age, use illegal drugs, and engage in illegal activities [41]. Research indicates that parental supervision and support is a key mechanism that links single-parent households with poorer outcomes in adolescence and young adulthood [42,43], and lower levels of parental monitoring is predictive of a variety of adverse outcomes including school truancy, substance use, drug trafficking, antisocial behaviors, sexual activity, and violent behaviors [44,45].

### 2.4. Improving Neighborhood and Housing Conditions

Given that virtually every health-enhancing resource is linked to where one lives in the U.S., a key to improving health and reducing disparities is to improve the quality of neighborhood and housing environments in the United States. There are multiple examples that illustrate that neighborhood transformation is possible and is associated with improved health. The Yonkers Housing Intervention was a city-wide de-concentration of public housing [46]. As a part of this initiative, half of public housing residents were selected via a lottery to move to better housing. Two years later, compared to those residents who had stayed, those who moved reported better health (overall health and lower levels of substance abuse), higher neighborhood quality (lower neighborhood disorder and violence and higher levels of satisfaction with public transportation, recreation facilities and medical care) and improved economic outcomes (higher rates of employment and lower rates of welfare use).

The Seattle-King County Healthy Homes Project was an intervention that improved housing conditions for low-income families. This program utilized community outreach workers to work with neighborhood residents to develop an intervention plan that was tailored to improve the safety and quality of their homes, as well as, reduce triggers for asthma [47]. A randomized controlled trial (RCT) with these low income households in which children with asthma were present found that the program was effective in reducing asthma symptoms and urgent health care use among the children, and in improving quality-of-life among the adults who cared for them [48].

The Harlem Children’s Zone (HCZ) is another example of a comprehensive neighborhood revitalization effort that sought to address several interrelated social determinants that affect the living conditions and health of children and their families, including quality early education, after school programming, access to health services, violence prevention initiatives, and multiple other community-level factors [49]. Rigorous evaluation of the HCZ has shown that it eliminated racial gaps in academic achievement and was successful in improving housing conditions and childhood asthma outcomes [50,51]. Based on the success of this program, the HCZ was a model for the Obama Administration’s Promise Neighborhood Initiative, a federal program that provided support for the development of 20 similar “promise neighborhoods” in the United States.

During the Clinton Administration in the 1990s, there were three major initiatives out of the federal Department of Housing and Urban Development (HUD) that sought to improve the economic opportunity and the quality of life in disadvantaged communities [52]. The first, Moving to Opportunity (MTO), focused on residential relocation. Its goal was to help poor families move from high-poverty public housing to neighborhoods with lower levels of poverty. The second, Jobs-Plus, sought to increase employment by providing in-place services and incentives for work. Concretely, this program sought to saturate public housing with high-quality employment services and rent-based financial incentives. The third initiative, Bridges to Work, sought to link central city residents to available jobs in the suburbs. Specifically, it focused on helping residents of high-poverty, central-city communities find jobs in suburban areas that were rich in employment opportunities.

Evaluation of these HUD programs showed that poor and minority families will respond and take advantage of real opportunities [52]. Moreover, these interventions can increase income, improve safety and security and improve physical and mental health. However, programs that will be effective in the long term must tackle all of the major barriers that poor individuals and communities face: housing, safety, health, employment and education. For example, people need help not only in finding jobs but also in keeping jobs. This means that issues of retention, advancement, commuting costs, and child care also have to be addressed. In addition, meaningful change requires sustained effort over time. There has been rigorous evaluation of the health impact of the MTO Program. One study found that 10 to 15 years after poor public housing families had been randomized to move to less poor neighborhoods, those who had moved had lower levels of obesity, severe obesity and diabetes risk [53].

Research also indicates that the benefits of moving poor residents from high-poverty neighborhood environments to lower poverty residential areas can co-occur with negative effects. For example, in both the Yonkers Housing Intervention [54] and Moving to Opportunity project [55], adverse educational and behavioral outcomes were observed among the teenagers who were older at the time of the initial move. This highlights the importance of early intervention and/or the need of minimal thresholds of exposure to the new environment before positive effects are evident. Research also reveals that moving poor residents out of their neighborhood disrupted their social networks. For example, in the Yonkers Housing Intervention, compared to poor residents who did not move, movers had weaker social ties in their new neighborhoods and smaller networks of neighborhood-based friends [56].

Thus, although the housing intervention studies provide high quality scientific evidence of the influence of neighborhood environments on health, the observed unintended negative effects suggest that moving poor persons out of their current neighborhoods may not be an optimal policy approach. Instead, emphasis should be on improving living conditions in poor residents’ current residential environments. Purpose Built Communities, based on its initial efforts in Atlanta’s East Lake district, has developed a model that seeks to transform existing neighborhoods by addressing all of the challenges faced by disadvantaged communities simultaneously [57]. This model views the geographic concentration of poverty as the critical issue to address and emphasizes the need for comprehensive, place-based solutions that work across traditional silos of education, housing, public safety, employment opportunity, child care and nutrition. The comprehensive and integrative strategies deployed include an independently run cradle-to-college educational system that provides high-quality early child development programs that attracts middle-income families but also ensures that low-income students start school ahead of grade level. Another key aspect of the model is quality mixed-income housing that de-concentrates neighborhood poverty while assuring affordability for low-income households. Other critical community services provided include employment support through a range of workforce development initiatives, recreational opportunities and other social services. Ten years after the implementation of this model in East Lake Atlanta, crime had declined by 73% and violent crime by 90% [57]. Similarly, employment among low-income residents increased from 13% to 70%, and the elementary school was transformed from one of the worst performing among Atlanta public schools to one of the best performing in the state, even though 74% of the students qualify for free and reduced price lunch. The purpose built model is being replicated in multiple communities across the U.S.

## 3. Strategy Number Two: Build more Health into the Delivery of Medical Care

It is estimated that although 75% of health care dollars in the U.S. is spent on preventable conditions, only 3% of America’s health care expenditure is on prevention [58]. Thus, a renewed emphasis on prevention could have an enormous impact on improving overall health and reducing inequities in health. There are multiple strategies that would enable the American healthcare system to have a greater impact in improving the health of all and reducing inequities in health care. These include ensuring access to care for all, giving greater emphasis to primary care over specialty care, eliminating inequities in the receipt of high quality care, addressing patients’ social risk factors and needs more effectively, and diversifying the healthcare workforce.

### 3.1. Ensuring Access to Care for All

Providing access to comprehensive preventive screenings and treatment can play a role in reducing and eliminating at least some racial inequities in health. A statewide colorectal cancer (CRC) initiative in the state of Delaware illustrates that a concerted effort can produce striking changes in a relatively short time [59]. The program began in 2002 and provided reimbursement for a colonoscopy for any uninsured Delaware resident with household income up to 250% of the poverty level. In 2004, a treatment program was added that covered the costs of cancer care for two years for all uninsured residents who were newly diagnosed, if their household income was up to 650% of the poverty level. In that same year a nurse navigator system was added to facilitate access to screening and treatment and special outreach efforts were made to black residents. By 2009, colonoscopy screening rates had increased by 54% for blacks and 29% for whites and the racial gap in screening was eliminated. Similarly, a 34% decline in CRC incidence for African Americans and a 26% decline for whites led to the equalization of the incidence rates. The mortality gap was almost eliminated by 2009 with a mortality decline of 42% for blacks and 13% for whites. Moreover, the program produced cost savings. The annual bill for the screening and treatment program was $7 million but the annual savings from reduced incidence of CRC was $8.5 million.

Initiatives are needed that ensure access to health care for all. Research revealed that the implementation of the Affordable Care Act (ACA) increased insurance coverage for 20 million Americans, reduced the gap between whites and nonwhites for all racial groups in the U.S., and completely eliminated that disparity for Asian Americans, Native Hawaiians and other Pacific Islanders, but not for other racial groups [60]. Efforts are needed to ensure access to care for the millions of Americans who still lack insurance. In 2017, Senator Bernie Sanders introduced such an initiative. It is called the Medicare for All Act and aims to provide a universal right to decent healthcare for all U.S. residents. A recent analysis of the costs of this program revealed that although it would increase the demand for healthcare services by about 12%, the implementation of the program could realize cost savings of about 19%, which would reduce overall healthcare costs by about 9.6% [61].

### 3.2. Emphasize Primary Care

Much of medical care in the U.S. is focused on treating disease after it has occurred. Research reveals that a greater emphasis on person-focused primary care (instead of disease-focused care) would better address the health care needs of patients and reduce inequities in health [62]. Access to primary care is associated with smaller social disparities in health and is a likely determinant of the unexpectedly good health profiles of countries like Cuba and Costa Rica [63]. Starfield and colleagues have reviewed the evidence on the contribution of primary care to health [63]. They have documented that for multiple indicators of health status, health is better in places with a higher ratio of primary care doctors to the population than in other areas, even after adjusting for demographic factors, SES and health behaviors. In addition, at the individual level, people who have a primary care provider rather than a specialist as their regular source of care are also healthier. As noted, health inequities are smaller in areas of greater concentration of primary care providers. Several aspects of primary care appear to contribute to this: primary care increases access of disadvantaged populations, provides an emphasis on preventive care and the early management of disease, encourages high quality care, more appropriate care and reduces unnecessary specialty care [63]. Moreover, studies indicate that a greater emphasis on primary care is also associated with lower total costs of health services [63].

### 3.3. Eliminating Inequities in the Receipt of High Quality Care

A 2003 report from the National Academy of Medicine (NAM; formerly, Institute of Medicine) concluded that across virtually every type of therapeutic intervention in the U.S., ranging from high technology procedures to the most basic forms of diagnostic and treatment interventions, blacks and other minorities receive fewer procedures and poorer quality medical care than whites [64]. Strikingly, these differences persisted even after statistical adjustment for variations in health insurance, SES, stage and severity of disease, co-occurring illness, and the type of healthcare facility are taken into account. More recent research documents the persistence of racial inequities in the quality and intensity of care [65,66]. For example, among adult first-time kidney transplantation candidates in the United States who were added to the deceased donor kidney transplantation waiting list between 1995 and 2014, disparities in the receipt of live donor kidney transplantation (LDKT) increased from 1995–1999 to 2010–2014 [67]. In 1995, the cumulative incidence of LDKT at 2 years after appearing on the waiting list was 7.0% among white patients, 3.4% among black patients, 6.8% among Hispanic patients, and 5.1% among Asian patients. In 2014, the cumulative incidence of LDKT was 11.4% among white patients, 2.9% among black patients, 5.9% among Hispanic patients, and 5.6% among Asian patients. From 1995–1999 to 2010–2014, racial disparities in the receipt of LDKT increased (*p* < 0.001 for all statistical interaction terms in adjusted models comparing white patients versus black, Hispanic, and Asian patients). Another example pertains to maternal mortality. Racial disparities in in-hospital maternal mortality decreased between 2006 and 2015, but significant disparities remain [68]. In 2006 the rate of in-hospital death was 248 percent higher for Black women, 50 percent higher for Hispanic women, and 69 percent higher for Asian/Pacific Islander women than for White women. In 2015, the rate for Blacks was 193 percent higher and the rate for Hispanics was 31 percent higher than the rate for Whites.

The NAM concluded that implicit bias on the part of healthcare providers was a likely contributor to these observed patterns [64]. Research reveals that these implicit biases are normal, natural, subtle and often subconscious [69]. Moreover, they are universal, all humans have them, with even the most well-meaning individual capable of harboring deep-seated biases. These biases are developed naturally through routine social interactions and exposure to culture (media, etc.). Importantly, they guide our expectations and social interactions with others and can become harmful when assumptions and generalizations about a group affect our interactions with an individual. Research also indicates that rapid and unconscious emotional and neural reactions to blacks occur for most Americans within 100 milliseconds, that is, in about one third of the time that it takes for us to blink our eyes (300 to 400 milliseconds) [70].

Analyses of data from a large, volunteer and non-representative sample of persons who took the Implicit Association Test (IAT), a widely used test to assess the presence of implicit bias, concluded that about 70 percent of physicians have an implicit preference for whites over blacks, similar to the pattern observed for other professionals and the general population [71]. Research reveals that higher implicit bias scores among physicians is associated with biased treatment recommendations in the care of black patients [66], although the pattern is not uniform [72]. This highlights the importance of research to better understand the conditions under which these biases are most likely to occur.

Implicit biases may affect not only medical decision making but also the quality of communication and nonverbal behavior [66]. One study found that black patients provided poorer ratings of the quality of the medical encounter (in terms of warmth, friendliness, teamwork and satisfaction) with physicians who were aversive racists (low on explicit bias and high on implicit bias) than with those who were high or low on both explicit and implicit racial bias [73]. Another study of providers who work in safety net clinics in a major metropolitan area found that provider implicit bias was associated with poorer quality of patient provider communication and lower patient evaluation of the quality of the medical encounter including provider nonverbal behavior [74]. For example, more implicit bias was associated with less patient-centered dialogue, lower patient positive affect, lower perception of respect from the clinician, less patient liking of the clinician and lower trust and confidence in clinician

Research is needed to identify optimal strategies of raising health providers’ awareness of subtle, unconscious discrimination and providing them with strategies to minimize its occurrence. One British study found that pharmacological intervention can reduce implicit bias. This randomized double blind, parallel group, placebo controlled study evaluated the impact of a single oral dose of propranolol (40 mg) in 36 whites [75]. Propranolol is a beta blocker that reduces emotional conditioning and amygdala responses to visual emotional stimuli (e.g., facial expressions). The study found that compared to placebo, propranolol eliminated implicit bias and reduced heart rate, but had no effect on explicit bias (measured by feeling thermometer: warmth towards blacks, whites, homosexuals, Muslims, Christians, drug addicts). While pharmacological interventions provide evidence that fundamental biological processes are present with the occurrence of implicit bias, they are not long-term practical solutions for effectively addressing implicit bias in the healthcare encounter.

There is emerging evidence that there are a broad range of social psychological interventions that are likely to reduce implicit bias among providers [76]. One study of university students documented that non-black adults could be motivated to increase their awareness of racial bias, their concerns about the effects of such bias and their willingness to implement strategies to reduce such bias [77]. These researchers viewed implicit biases as deeply engrained habits that can be replaced by learning multiple new prejudice-reducing strategies including stereotype replacement, counter-stereotype imaging, individuation, perspective taking and increasing interracial contact. The study found that these strategies reduced bias in participants and that this effect remained evident three months later. Future research is needed to assess the extent to which these changes in implicit prejudice are associated with the actual reduction of discriminatory behavior and the extent to which these programs can be effectively implemented on a large scale among healthcare providers.

Although implicit bias is likely the most common form of bias among healthcare providers, explicit bias also persists in society and efforts are needed to minimize its occurrence as well. Relatedly, there is also growing concern about the need to address structural competence among healthcare providers [78,79]. The term structural competence refers to increasing awareness among providers of the ways in which racism is embedded in our culture and institutions and shapes not only behavior at the individual level but also the ways in which policies, and procedures in medical and other social institutions, have initiated and sustain racial inequality. Accordingly, effectively addressing implicit bias requires identifying and dismantling its institutional legacies and social consequences. This will require changes not only in the individual behavior of providers but also policy changes across multiple domains of healthcare and other social institutions.

### 3.4. Addressing Patients’ Social Risk Factors and Needs

Another strategy for putting more health into the delivery of health care is for hospitals and other health care providers to be proactively engaged in connecting patients with supportive social services that will help them to improve their health. The Medical Legal Partnership (MLP) is a program that was developed in the Pediatrics department at the Boston Medical Center over three decades ago that addresses the social determinants of health [80]. MLP enabled primary care providers to refer patients to a new category of specialists: on-site attorneys. The program is premised on the idea that most low-income persons face legal issues that affect their quality of life and their management of disease. For example, all the asthma medications in the world will not enable a child with asthma to breathe symptom free if the underlying poor housing conditions that trigger the asthma in the first place are unaddressed. The addition of lawyers to the medical team facilitates screening families for, and assisting them with, problems that can affect effective care and illness management. The stressors addressed include challenges in the areas of housing, immigration, income support, food, education access, disability and family law. The MLP program is now at hundreds of health care sites in the U.S.

Health Leads (HL) is another innovative program that places undergraduate student volunteers in the waiting rooms of hospital clinics or health centers [81]. They assess patients’ needs regarding food, housing, heating or other social issues. They then “fill” the prescription for food assistance, employment or housing improvement by connecting patients to local resources through in-person meetings or telephone calls. A study of 1059 low-income families at a pediatric clinic found that the most prevalent needs for families were in employment (25%), housing (14%), child care (13%), health insurance (11%) and food assistance (10%). Within six months of contact with HL desk, half of the families had received help from at least one community resource [81]. HL is currently in multiple waiting rooms of hospital clinics and health centers across the U.S.

The Nurse-Family Partnership (NFP) program is an innovative early childhood intervention that seeks to address the health and social needs of mothers and their infants within the health care system [82]. In the NFP, nurses make home visits to low-income, first-time mothers. The visits begin during pregnancy and continue after the baby is born. The visits take a comprehensive view of the mother’s life and seek to improve maternal and child health, as well as address future life opportunities and economic self-sufficiency for the mothers and enable them to provide nurturing and competent childcare [82,83]. The care delivered through three RCTs (one in upstate New York with predominantly white women, one in Memphis, Tennessee with predominantly African American women and one in Colorado with predominantly Latinas) have documented that the program has positive effects for both parent and child [18]. Mothers in the control group received traditional prenatal care so the NFP documented the effects of the additional services provided by the program. For mothers, the NFP led to lower smoking during pregnancy, fewer subsequent pregnancies, increased labor force participation, reduced use of public assistance programs, and lower rates of child abuse and neglect. Among the children, the program led to a reduction in childhood injuries, substance use and juvenile crime [82,84]. An evaluation of the three NFP trials estimated that the program saves $18,054 for each family served [85].

The Community Aging in Place, Advancing Better Living for Elders (CAPABLE) program uses an inter-professional team (an occupational therapist, a registered nurse, and a handyman) to help low income older individuals with self-care disabilities achieve functional goals they set [86]. A demonstration project conducted in 2012–2015 revealed that 75 percent of participants had improved their performance of activities of daily living over a five-month period. In an RCT of the program, 79% of participants improved [87]. There were significant improvements in activities of daily living (ADLs) and independent activities of daily living (IADLs) and reductions in depressive symptoms. The program cost less than $3000 per participant and provided a 10-fold return on investment via reduced healthcare utilization.

Hospitals are often the largest employer in many communities. Health care systems and other healthcare providers can make effective use of local community resources and strengthen their surrounding communities by providing job training and job opportunities (e.g., as community health workers or medical assistants) in *health* care to community residents with limited educational attainment that constrains their economic prospects. Such initiatives can help to improve the economic security, stability and health of people in low income communities while simultaneously addressing a growing need for health care workers.

The integration of community health workers (CHW) into the delivery of healthcare is another promising strategy. CHWs are health workers who have received formal but limited training and work to improve community health outside of healthcare facilities. Research over the last 25 years has shown that these workers can improve community health in low-, middle, and high income countries [88]. Reviews of research in the U.S. indicate that CHW interventions have been effective in improving the control of high blood pressure and reducing cardiovascular risk, enhancing diabetes control, managing HIV infection, and increasing the uptake of cancer screening tests [88]. A partnership between the National Institutes of Health (NIH) and the Patient-Centered Outcomes Research Institute (PCORI) has funded two pragmatic trials testing care models integrating community health workers into primary care teams to reduce disparities in hypertension control in racial minority and rural populations [89]. These studies also aim to elucidate and address barriers to implementing these care models in clinics caring for patients from underserved communities.

A panel of the NAM has highlighted the enormous potential of the routine screening of all patients for the social determinants of health, as a part of comprehensively addressing the needs of patients [90]. It has recommended social and behavioral factors that should be captured in the electronic medical record (EMR). In addition to race/ethnicity and education, it calls for the inclusion of brief indicators of the following factors: financial strain, stress, depression, physical activity, tobacco use, alcohol use, social ties, intimate-partner violence, current residential address and census-tract median income (geocoded). A committee of the National Academies of Sciences, Engineering, and Medicine is currently examining the potential for integrating services addressing social needs and the social determinants of health into the delivery of health care to achieve better health outcomes and to address major challenges facing the U.S. health care system, including healthcare disparities [91]. The committee will make recommendations on how to: (1) expand social needs care services; (2) better coordinate roles for social needs care providers in interprofessional care teams across the continuum of clinical and community health settings; and (3) optimize the effectiveness of social services to improve health and health care. Recommendations may address areas such as integration of services, training and oversight, workforce recruitment and retention, quality improvement, research and dissemination, and governmental and institutional policy for health care delivery and financing.

### 3.5. Diversifying the Healthcare Workforce

Considerable research suggests that developing a healthcare workforce that is reflective of the racial diversity of the population that it serves is in the best interest of the performance of the entire health care system [92,93]. This research indicates that underrepresented minorities (URM) are more likely to work with underserved populations, with URM from the highest SES backgrounds working in under-served areas at higher rates than do white physicians from the lowest SES backgrounds [93]. Research also indicates that racial concordance between a patient and a clinician has been associated with better patient-provider communication and overall health outcomes, as well as higher levels of patient satisfaction with care and adherence to provider recommendations [92,93]. Thus, as the U.S. population becomes increasingly diverse, with the current URM populations becoming the majority of the U.S. population in less than 25 years, ensuring the increasing diversity of health care providers is in the best interest of national health care delivery. 

A broad range of affirmative action policies have been implemented in the last 50 years to increase the participation and success of women and minorities in education and occupational contexts. It is insufficiently appreciated that affirmative action was much more effective for women than for minorities. For example, females graduates from medical school (most of them white), increased from 6.9% (n = 524) in 1965-66 to 46.3% (n = 8724) in 2015-16 [94]. In contrast, black, Latino and Native American graduates have had much smaller increases. For the 1968-69 school year, of the 9,863 first year medical school enrollees in the U.S., 266 (2.7%) were black, three (0.0%) were Native American, 20 (0.2%) were Mexican American and 3 (0.0%) were mainland Puerto Rican [95]. In 2015, Asians were 19.8% (3701), African Americans were 5.7% (n = 10,610) and Latinos were 4.6% (n = 854) of medical school graduates [96]. Only 20 (0.1%) American Indian or Alaska Natives and 5 Native Hawaiian and other Pacific Islanders graduated from medical school in 2015. Among URM graduates in 2015, females were the majority. Women made up 65% of black graduates, 53% of Hispanic graduates, 75% of Native American graduates and 60% of Native Hawaiian and other Pacific Islander graduates [96]. Stunningly, an American Association of Medical Colleges (AAMC) report indicated that in 2014, there were 27 fewer African American males in the first year of Medical School than in 1978 [97]. In the mid- 1960s, 2.9% of all practicing physicians in the U.S. were black, and in 2012, 3.8% of all practicing physicians were black (5.2% were Hispanic) [98]. These data highlight that it is not enough just to open the doors of opportunity. Everyone, irrespective of social group and background, must have the ability to walk through those doors.

Although legislation, including the Minority Health and Health Disparities Research and Education Act of 2000 and The Affordable Care Act have included provisions and authorized funding to encourage workforce diversity in the health professions, many of the programs that were established are at risk for elimination with reductions in funding or changes in authorization of funding [99]. Federal funding should be increased to support the recruitment and retention of students and faculty from URM backgrounds and health professionals—especially physicians—from diverse racial and social class backgrounds, to practice in medically underserved urban and rural areas. This funding can be channeled through the NIH, the Indian Health Service, the Centers for Disease Control Office of Minority Health, and the Health Services and Resources Administration (Title VII and VIII Health Professions Training Grants, National Health Services Corps, Centers of Excellence Program, reauthorization of the Health Careers Opportunity Program (HCOP), the Scholarships for Disadvantaged Students program, Nursing Workforce Diversity Program, and grants to support the Community Health Workforce). These programs should include, but not be limited to, scholarship and loan repayment programs and institutional resources to increase diversity. Programs should include outreach, mentoring, and tutoring at all educational levels—including elementary and high school and college—to encourage URM students to pursue careers in science and health. Federal and state legislation should support the consideration of race, family income, and first generation college graduate status in determining admission to institutions of higher education.

## 4. Strategy Number 3: Raising Awareness of Inequities and Building Political Will to Address them

There are three interrelated communication challenges: we need to raise awareness of inequities, build political support, and increase empathy for addressing social inequities in health.

### 4.1. Increasing Awareness that Racial Inequities Exist

We need to raise awareness levels of the problem of racial inequities in health. Data from prior national surveys indicate that the majority of American adults were unaware of gaps in health between African Americans and whites [100]. More generally, Americans markedly overestimate progress that has been made toward racial economic equality [101]. A recent study found that the degree of current racial equality (e.g., in income, wealth and wages) was overestimated by about 25% and was most pronounced among high-income whites and was strongly associated with beliefs in a just world [101]. Research also reveals that Americans do not naturally think about health in terms of social factors. For example, in national data, 84% of Americans view their health as largely under their control and for which they have to take personal responsibility and most of the U.S. public view health behaviors and access to care as very strong determinants of health [102]. Many fewer see social and economic determinants as having strong effects on health, with older, non-white, liberal and low SES individuals being much more likely to see social and economic factors as important.

Narrative approaches are needed to contextualize the social determinants of health and help advantaged groups envision and sympathize with the harsh realities of disadvantaged contexts. Effective communication is also needed to balance attention to personal responsibility for health with simultaneous attention to social responsibility that seeks to remove the barriers and create the opportunities that would ensure that everyone has access to opportunities to be healthy. The existing research on the effective strategies for raising awareness of racial inequities in health by using the mass media is limited and significant gaps exist in our current understanding [100]. We need more systematic and sustained attention to identifying the optimal communication strategies to effectively communicate the avoidable burden of disease, disability and premature death caused by health inequities and their social and economic costs at the local and national level.

The challenge of raising awareness levels has markedly increased in recent years because the U.S. and at least some other Western nations have moved into what some observers have called a “post-fact” and “post-truth” world [103]. Key characteristics of this new era include a brazen disregard for facts driven by rejection of experts and a distrust of what is presented as fact, especially if it is uncomfortable. What counts as a fact is simply what the individual feels to be true. In this context, anyone can make up opposing (and even deceptive) facts. In this environment, outrageous claims and behavior become normalized and on many social issues large segments of the population appear to be “a misguided mob” instead of “an informed public”. Research is needed to identify how, in this current environment that is anti-elite, anti-authority and has little trust in social institutions, credible voices can be established that all will accept as authoritative, even if there are varying perspectives of how best to effectively address the underlying issue.

### 4.2. Building Political Support to Address Inequities

Public opinion research over the last four decades in the U.S. has shown that levels of support for government interventions to help blacks have been low and they are declining over time [104]. Moreover, Americans show more support for income or class-targeted policies than for racial ones. For example, in national data, 70% of whites would support giving businesses and industry special tax breaks for locating “in poor and high unemployment areas” but only 43% of whites would support such initiatives in “largely black areas” [104].

Communication research reveals that many Americans view racial inequities through framings that block their willingness to support effective action. Research by Frameworks Institute [105] has documented how Americans think about racial disparities. These dominant frames include that American society has made important progress on race in recent decades because changes in laws and policies have eliminated the discrimination and racism that had characterized American society in the past. There is a residual racism but it is only at the individual level and it is as common in minorities as in whites. Accordingly, discrimination plays no role in racial inequities, and any differences in societal outcomes between whites and non-whites are due to blacks’ failure to live by core American values such as those linked to personal responsibility, self-reliance, and effort. Importantly, these dominant frames and cultural narratives about race are activated when racial disparities are mentioned. A national survey conducted in the fall of 2018 found that these beliefs are endorsed by a substantial proportion of Americans [106]. Forty two percent of adults (45% of whites, 43% of Hispanics and 24% of blacks) indicated that blacks would do as well as whites if they worked harder. There is also considerable partisan polarization on these beliefs with 72% of Republicans compared to 23% of Democrats reporting that social disparities are due to blacks not trying hard enough.

Research by Frameworks Institute has also identified the type of approaches that are likely to be successful in building support to address racial inequities. Based on testing of a variety of framings that could be used to talk about disparities, the researchers concluded that many widely used framing strategies do not work because the dominant racial framing blocks this alternative viewpoint. These ineffective approaches included framing diversity as a strength, arguing that disparities for minorities were canaries in a coal mine (early warning indicators), or framing racial inequities as reflecting white privilege or as structurally driven [105]. Framings identified as likely to be successful in building support to address disparities included those that de-emphasized racial inequities but concentrated on widely shared American values (like ingenuity and enhancing opportunity for all) and that linked communities in a sense of shared fate. Specific frames identified included emphasizing opportunity for all, highlighting effective solutions and innovation, making salient the interdependence of all communities, stressing preventing community problems before they occurred, and emphasizing fairness (not between individuals but) between places have the potential to be successful. These findings highlight the need to build the science base that will guide us in developing the optimal ways of framing core equity principles and identifying the specific framings that would be most effective in building the political will and enhancing the power of historically disadvantaged groups to effectively address racial and SES inequities in health.

### 4.3. Increasing Public Empathy

Relatedly, research is needed to identify how to tell the story of the challenges of the disadvantaged in ways that increase empathy and emotionally connect and resonate with the public. Empathy is viewed as having two components: (a) cognitive empathy which refers to the ability to take the perspective of the other, and (b) affective empathy which is the ability to experience the emotion of others [107]. In his 1899 book on the Philadelphia Negro, W.E.B. DuBois described America’s lack of empathy for racial inequities in health as “the peculiar attitude” that was “the most difficult social problem in the matter of Negro health” [108]. He elaborated that “There have… been few other cases in the history of civilized peoples where human suffering has been viewed with such peculiar indifference” [108] p.163.

A large body of research documents that there is a racial gap in empathy, in which individuals have empathic responses to members of their own group but not to members of a racial outgroup [109]. Some of the strongest evidence comes from studies that assess empathic responses in brain activity when viewing the suffering of individuals of one’s own race versus that of members of another race. A recent review documents that a racial ingroup bias in empathy has been consistently documented in brain imagery studies in Europe, Africa, Asia, and the United States [110]. This research indicates that there is a stronger empathic neural response to the pain of same race versus other race individuals, using a variety of stimuli, and this racial bias in neural responses is more consistent than self-reported estimates of empathy. This research has also documented that the racial ingroup bias is more pronounced among persons with higher implicit racial bias, individuals from cultures that are more collectivistic than individualistic in emphasis, and when individuals are exposed to harsh living conditions [110]. Also, the experimental administration of oxytocin increases empathic brain activity to same race persons but not to persons of another race.

A study measuring EEG brain activity of non-blacks illustrated how showing less empathy for members of racial outgroups appears to be deeply embedded in the brain [111]. It found that seeing someone from one’s own racial group who is sad elicited the same brain activity as feeling sad oneself. In contrast, viewing someone of another race (outgroup), triggered no corresponding brain activity. Consistent with the larger literature, the more prejudiced the person, the less activity was evident (felt distress) when viewing the outgroup member. There was no difference by specific outgroup (white, black, South Asian, East Asian). Thus, people are less likely to ‘catch and match’ the emotion of outgroup members and therefore less likely to be motivated to act to meet their needs.

Research also indicates that a lack of empathy develops early in life [112]. For example, a study of mainly white 5-, 7- and 10-year olds had them rate the pain that they thought target black and white children had experienced after being stuck by a needle. In all scenarios, the action of inflicting pain was identical, only the target of the pain varied. The study found that there was no racial bias at age 5. However, children showed a weak bias (blacks feel less pain) at age 7. But by age 10, there was a strong, reliable racial bias with children rating the pain of the black child less than the white one. This highlights the need to start empathy training very young.

Research has also identified the conditions under which the racial gap in empathic brain activity is reduced [110]. These include when individuals are instructed to focus on an individual’s suffering (individuation) instead of his/her racial background, when individuals are on the same team with members of a racial outgroup, and among persons who have experienced greater inter-racial interaction in their socialization. Major investments in research are needed to identify how to best close the empathy gap in real world contexts and to expeditiously bring to scale interventions that are proven to be effective.

Relatedly, research indicates that emotions have a large impact on decision-making in general, and on race-related attitudes and policy, in particular. Thus, the absence of positive emotions for stigmatized groups can shape social policies that can affect their socioeconomic opportunities and health. This absence of positive emotions has been identified as an important component of subtle racial prejudice [113], and research documents that the lack of positive affect towards an outgroup is a strong predictor of discriminatory behavior and policy preferences. A meta-analysis found that emotional prejudice was twice as strongly predictive of discriminatory behavior as racial beliefs and stereotypes [114]. A study in Germany, the Netherlands, France and the U.K found that the absence of positive emotions (measured by two items that captured the lack of feelings of sympathy and admiration towards the outgroup) was a strong predictor of opposition to policies regarding immigrant outgroups [113]. Similarly, research in the U.S. has found that the same measure of the absence of positive emotions for blacks was the strongest predictor of whites’ opposition to affirmative action in employment and to an active role of the government in reducing racial inequalities [115]. Other research reveals that racial prejudice was a driver of the opposition to President Obama’s health care reform legislation with the racial divide in attitudes toward health care being 20 percentage points larger in the Obama era than it was in the early 1990s when Americans were considering President Clinton’s plan [116].

### 4.4. Enhancing Individual and Community Capacity

Building political will requires deliberate and explicit initiatives to embrace and strengthen the capacity of community residents and various community institutions (families, neighborhoods, schools, churches, businesses and voluntary agencies) so that they are empowered to be actively engaged in solving community problems. Research has found that those most knowledgeable about social factors are often the least politically active [102]. Importantly, community needs can be more effectively addressed when local institutions and stakeholders can be enlisted to be agents of change to seek solutions to local problems. Community-based interventions can view the community as the target of change with a goal of creating a healthier community through changes in community policies, environments, institutions and services [117,118].

Importantly, attention should be given to ensuring that both community residents and institutions receive needed knowledge and technical skills to maximize the potential impact of interventions. For example, in the Great Smoky Mountains Study, the provision of additional income was associated with increased adolescent obesity among low-income families, but there was no effect among high-income families [30]. These findings suggest that low-income families could have benefited from consumer information that enabled making healthier nutritional choices that could have maximized the health impact of increased expenditures on food. An intervention that targeted low-income black and white parents to start a college education fund for their child in Head Start also illustrates the need to empower consumers with information [119]. In this project a parent’s deposit of $25 would provide $1000 in the child’s account, and any additional deposits by the parent would be matched dollar for dollar. However, only 62% of the black parents and 67% of the white parents enrolled in the program. An evaluation revealed that these parents had low levels of financial literacy, a history of negative experiences with financial institutions and more severe household financial constraints than was recognized by the designers of the intervention [119]. Thus, a successful intervention requires adequate awareness and empowerment on the part of the targeted group.

Efforts are also needed that provide support for mental health treatment and promote psychological well-being. Research finds, for example, that mental disorders are more disabling that many common chronic conditions such as diabetes, arthritis, cardiovascular disease and cancer [120]. Good mental health is more than the absence of disease. Thus, in addition to providing access to treatment for mental disorders, attention should also be given to community wide initiatives to promote positive psychological resources such as optimism, purpose in life, personal mastery and positive relations with others. There are examples of comprehensive mental health interventions at the individual and community level that have improved access to a broad range of mental health and substance use services in communities that were historically underserved [121,122].

Enhancing individual and community capacity also requires increased attention to building racial pride as an explicit strategy for mitigating some of the negative effects of the devaluing of stigmatized groups that is deeply embedded in the pervasive negative ideology about marginalized racial groups that is part of the larger culture. A small but growing body of research suggests that such initiatives can enhance health. A study of 16 to 18 year old African American and Latino males being released from jail randomized them either to the standard single session of jail-based, discharge planning or to an eight session, 30-hour educational program that started in jail but continued after release. These sessions addressed issues of drugs, sexual relationships and risk factor reduction, but also explicitly devoted time to building racial pride. The study found that one year after release, those who had received the comprehensive intervention had spent fewer days in jail, were more likely to have attended school and found work, and had reduced odds of substance abuse [123]. Follow-up analyses revealed that there was an inverse association between racial pride and substance abuse, violence, recidivism, and a positive association with education and employment after being released from jail [124]. Similarly, a RCT with African American girls aged 14 to 18 built gender empowerment, racial pride and solidarity with the black community into the four 4-hour sessions. The study found that the intervention enhanced HIV-prevention behaviors and skills such as increased condom use 6 months and 12 months after the intervention [125,126]. More generally, a growing body of research indicates that values affirmation interventions that enhance stigmatized racial group members’ sense of self-worth are associated with improved academic performance, health and health behaviors [127].

### 4.5. Dismantling Racism

It was indicated earlier that developing communities of opportunity requires dismantling the negative effects of institutional racism. Building political will and support for the long term will also require efforts to eradicate cultural racism—the ideology of the inferiority of some racial groups that is pervasive in the norms, values, symbols, language and assumptions of the larger culture and that shapes the attitudes, values, stereotypes and behaviors of many individuals in society [9]. This is a necessary step to reduce prejudice, and end discrimination at the societal level. This may be an especially challenging task in the current political environment in which racial attitudes and values that may have been deep-seated and dormant have now surfaced and flourished in ways that are driving politics and policy [128,129,130], and where there is increased salience and visibility of incidents of racial prejudice and discrimination.

There is growing evidence that racial prejudice and resentment is one of the critical determinants of the appeal of Donald Trump to a substantial proportion of American adults [128,129,130]. One study found that after adjusting for a broad range of other factors, whites who strongly agreed with the statement that they were afraid of people of other races were ten times more likely to have voted for Trump compared to those who disagreed [128]. Similarly, reminding white Americans who scored high on a measure of racial identification that whites would become a minority of the U.S. population in 2042, led them to be more concerned about the declining power of whites in the U.S. and to report increased support for candidate Donald Trump and for anti-immigrant policies [131]. Another study using two national surveys assessed the relative contribution of concerns about economic well-being compared to racial attitudes and sexism to support for Donald Trump’s candidacy and the education gap in support for him [129]. Among whites, Trump had a four point margin of voter support over Hilary Clinton among college graduates, but a nearly 40% margin among those without a college education. The analyses found that a denial of racism and a hostile sexism, rather than economic difficulties, were the key drivers of support for Trump, especially among whites with low levels of education. For example, in one of the surveys, as respondents moved from a high level of acknowledgement of racism to a denial of racism, there was a 60-point increase in support of Trump. Similarly, moving from non-sexist views to sexist ones was associated with a 20 percentage point increase in support of Trump [129]. These associations were evident after holding constant multiple factors that predict voting behavior. Also, denial of racism more strongly predicted voting behavior in 2016 than in 2012 and it predicted some voters who voted for Obama in 2012 becoming Trump supporters in 2016. An experimental study also found that simply exposing white Trump supporters to a picture of a black (vs. a white man) in the context of seeking their support for a housing assistance policy, led them to be less supportive of housing assistance programs, angrier that some people receive assistance from government, and more likely to blame people who receive assistance for their circumstances [132].

At the same time, there is also emerging evidence that relatively simple interventions can reduce prejudice and discrimination. A recent rigorous study documented that prejudice (in this case, against transgender individuals) can be reduced with a 10-minute non-confrontational conversation [133]. In this study, in the intervention group, 56 canvassers went door to door encouraging 501 voters to take the perspective of a transgender individual, using a script in which the voter did most of the talking. The canvassers’ job was to enable voters to recall a time when they had been treated negatively so that they would understand the perspective and empathize with the challenges faced by a transgender person. Voters in the control group had a conversation with the canvasser about recycling. The study found that this intervention led to substantial reductions in prejudice against transgender people and increases in support for a law that would protect transgender individuals from discrimination. Importantly, these changes were present for Democratic and Republican voters and conversations were effective with both transgender and non-transgender canvassers. Moreover, the effects were still evident three months after the intervention. Future research is needed to replicate this strategy with racial outgroups. Importantly, the intervention script emerged from the systematic testing of the relative persuasiveness of more than 13,000 canvassing conversations, highlighting the important work that is needed in testing alternative communication strategies [134].

Similarly, an RCT in Japan showed that it was possible to get Japanese university students to increase their empathy toward a stigmatized outgroup (Japanese-Brazilians residing in Japan who had lost their jobs because of an economic recession) [135]. In this study the intervention group read an emotional essay which was designed to help participants feel anger about the challenges that Japanese-Brazilians face in Japan and to feel sadness about their suffering. The control group read an objective essay that provided factual information about the migration of Japanese-Brazilians to Brazil. Both groups either wrote a letter to a member of the immigrant outgroup or about an environmental issue facing Japan. The study found that the emotional essay increased feelings of anger about the problems faced by Japanese-Brazilians and sadness about their distress. The study also found that increases in empathy were much larger among persons who read the emotional essay and wrote a letter than in any of the other combinations of conditions. Empathy levels were still elevated a week later. This suggests that establishing emotional connection along with perspective taking enhanced empathy.

Research is also needed to identify the optimal interventions for reducing racial prejudice and ideologies at the societal level. A study in Rwanda found that a year-long experimental intervention that used a radio-based soap opera that incorporated cultural songs and traditions was successful in producing modest reductions in tribal prejudice and conflict [136]. Similarly, mass media interventions in the U.S. have played a role in reducing prejudice and stereotypes toward gays in recent decades [137,138]. These have generally involved the sympathetic portrayal of gay characters on television. More research is needed to identify how greater use can be made of the mass media to reduce racial prejudice at the societal level and to identify the specific strategies and framings that would be optimal for reducing of racial prejudice for various segments of the population. The successful reduction of racism in the long term requires redoubled efforts to reduce and eliminate the racism that is deeply ingrained in the larger culture.

## 5. Opportunities

The evidence reviewed here indicates that while reducing racial inequities in health is neither simple nor straightforward, there is a sizable body of scientific research, much larger than most researchers and practitioners recognize, that has identified key priorities for comprehensive efforts to reduce and ultimately eliminate disparities in health. There is much that we need to learn about the optimal timing and sequencing for all of the identified interventions and the most promising ways to scale up interventions that have shown promise in small local studies. However, one of our society’s greatest needs is to make a resolute commitment to reduce racial inequities in health and to apply, in a systematic and sustained way, all of the evidence that we currently have.

## 6. Conclusions

Eliminating racial inequities in health requires three coordinated strategies. First, integrated efforts are needed to neutralize the ways in which racism truncates or blocks access to avenues of success by creating new place-based opportunities and enhancing existing ones to ensure that all individuals have access to the resources necessary to thrive. Second, initiatives are required to shift the health care system from a narrow focus on treatment to emphasize preventing disease and providing timely, appropriate, high-quality care for all that is tailored to the culture and context of each patient. Finally, given the limited awareness of the existence of racial inequities in health and the lack of commitment to address them, new investments are needed to create the knowledge base to identify the optimal strategies that would enhance awareness of the nature and extent of racial inequities and build the needed empathy and political will to eradicate them.
